# Ultrasound Assessment of Breech Engagement: Breech Progression Angle and Prediction of External Cephalic Version Success

**DOI:** 10.3390/jcm14207179

**Published:** 2025-10-11

**Authors:** Javier Sánchez-Romero, Rosa María Gallego-Pozuelo, Cristina Ortuño-Hernández, Ana Martínez-Zarco, Rocío Barroso-Linares, Fernando Araico-Rodríguez, José Eliseo Blanco-Carnero, Aníbal Nieto-Díaz, Catalina de Paco-Matallana

**Affiliations:** 1 Maternal-Fetal Unit, Department of Obstetrics and Gynecology, ‘Virgen de la Arrixaca’ Clinic University Hospital, 30120 Murcia, Spain; javier.sanchez14@um.es (J.S.-R.); rosagallego-96@hotmail.com (R.M.G.-P.); cristinaooh@gmail.com (C.O.-H.); anamartinezzarco@gmail.com (A.M.-Z.); rocio99barroso@gmail.com (R.B.-L.); araicos33@hotmail.com (F.A.-R.); eliblanco@um.es (J.E.B.-C.); anibal.nieto@um.es (A.N.-D.); 2 Department of Obstetrics, Gynecology, Pediatrics and Surgery, School of Medicine, University of Murcia, 30120 Murcia, Spain; 3 Biomedical Research Institute of Murcia IMIB-Arrixaca, 30120 Murcia, Spain

**Keywords:** breech progression angle, transperineal ultrasound, external cephalic version, buttocks, cesarean section, breech presentation

## Abstract

**Objectives**: To evaluate the role of breech progression angle (BPA), a novel transperineal ultrasound parameter, as a predictor of external cephalic version (ECV) success, and to compare BPA between breech and transverse lie presentations. **Methods**: This prospective exploratory study was nested within a randomized clinical trial (NCT06449430) at Virgen de la Arrixaca University Hospital, Murcia, Spain. Eligible participants were pregnant women ≥18 years with a singleton fetus in non-cephalic presentation at term, without contraindications to vaginal birth. BPA was measured transperineally following standardized methodology prior to ECV, performed under either spinal analgesia or propofol sedation. Logistic regression models adjusted for maternal and obstetric variables assessed the association between BPA and ECV success. Receiver operating characteristic (ROC) curves were generated to evaluate predictive accuracy. **Results**: A total of 117 women were included: 100 with breech and 17 with transverse lie presentations. Median BPA was significantly higher in breech compared with transverse lie (87.2° vs. 70.2°, *p* < 0.001). In the overall cohort, BPA was not significantly associated with ECV success (OR 0.97, 95% CI 0.94–1.00; *p* = 0.068). However, in breech presentations, BPA was independently associated with ECV success (adjusted OR 0.95, 95% CI 0.91–0.99; *p* = 0.015). The area under the ROC curve for BPA predicting ECV success in breech cases was 0.64 (95% CI 0.53–0.73). Predictive accuracy was poor for transverse lie (AUC 0.27, 95% CI 0.08–0.56). **Conclusions**: BPA measured by transperineal ultrasound does not provide clinically useful information for predicting the success of external cephalic version, either in breech or transverse lie.

## 1. Introduction

Non-cephalic presentation occurs in 3–5% of term pregnancies [1]. External cephalic version (ECV) is an effective procedure for converting fetal position to cephalic, thereby enabling vaginal birth with a lower risk than breech delivery or cesarean section [2].

Several interventions, such as tocolysis [3], sedation [4], or neuraxial analgesia [5], have been shown to increase the success rate of ECV. In addition, maternal and fetal characteristics—including multiparity, lower body mass index (BMI), placental location, amniotic fluid index (AFI), estimated fetal weight (EFW), and the degree of breech engagement—are associated with improved likelihood of cephalic presentation after the procedure [6].

In recent years, ultrasound has been explored as a tool for predicting ECV success [7]. Certain sonographic features, such as transverse lie, anterior placental location, higher AFI, unengaged breech, or anterior fetal back, have been associated with modest improvements in success rates [7]. However, assessment of breech engagement has traditionally relied on subjective evaluation, classifying the buttocks as engaged or unengaged based on clinical judgment [8,9,10].

For cephalic presentations, this limitation was addressed with the introduction of the angle of progression (AoP), a reproducible ultrasound measurement that has proven useful in assessing fetal head engagement and predicting vaginal delivery [11,12,13,14]. By analogy, the breech progression angle (BPA) has recently been proposed as an objective sonographic parameter to quantify breech engagement in the maternal pelvis [15]. Preliminary studies demonstrated that BPA can be reliably measured, but its clinical utility as a predictor of ECV success remains uncertain [15,16].

The primary aim of this study was to evaluate BPA as a predictor of ECV outcome in women with non-cephalic presentations at term. A secondary objective was to compare BPA measurements between breech and transverse lie presentations prior to ECV. By addressing these aims, we sought to determine whether BPA could provide clinically relevant information for predicting ECV success and guiding the management of breech presentation at term.

## 2. Materials and Methods

This was an exploratory prospective study embedded within the PropoSpinECV randomized clinical trial (RCT). The trial was conducted at the Department of Obstetrics and Gynecology, Virgen de la Arrixaca University Hospital, Murcia, Spain, and compared spinal analgesia with propofol sedation for ECV (ClinicalTrials.gov ID: NCT06449430) [17]. For this study, patient recruitment took place from 4 July 2024 to 1 July 2025. The protocol was approved by the local Research Ethics Committee (Approval Code: IMIB-ECV-2024-01. Approval Date: 20 May 2024), and all participants provided written informed consent. The study was conducted in accordance with the principles of the Declaration of Helsinki.

The assessment of the breech progression angle was pre-specified as an exploratory secondary objective within the randomized clinical trial PropoSpinECV. As the primary endpoint of the trial focuses on comparing spinal anesthesia and propofol sedation for ECV success, no specific sample size calculation was performed for BPA-related analyses.

### 2.1. Participants

Eligible women were ≥18 years old, with a singleton pregnancy in non-cephalic presentation at term and a desire for vaginal birth. Exclusion criteria included multiple gestation, risk of fetal compromise or active bleeding, contraindication to vaginal delivery (e.g., placenta previa), ≥2 prior cesarean sections, history of myomectomy with uterine cavity entry, maternal fever or thrombocytopenia (<85,000 platelets), allergy or intolerance to study medications (propofol, bupivacaine, fentanyl, ritodrine), ruptured membranes, established labor, fetal distress, or known fetal malformations or genetic disorders. Participants were identified during routine third-trimester ultrasound (35–36 weeks).

### 2.2. Ultrasound Assessment

On the day of the procedure, patients underwent a pre-procedure ultrasound to assess fetal position, biometry, amniotic fluid volume, breech progression angle (BPA), and fetal well-being. BPA was measured according to the technique described by Youssef et al. [15]. Women were examined in lithotomy position with an empty bladder. Transperineal ultrasound was performed using a Voluson S6 system (GE Healthcare, Zipf, Austria) with a convex 4–8 MHz probe covered by a sterile sheath was used. The probe was placed transperineally in the midsagittal plane, ensuring visualization of the pubic symphysis, urethra, vagina, and the presenting fetal part (breech or foot). BPA was defined as the angle between: (1) a line drawn along the long axis of the pubic symphysis, and (2) a second line from its most caudal point tangentially to the lowest identifiable portion of the fetal breech or foot.

Both 2D and 3D acquisitions were obtained. First, BPA was measured directly in real time on 2D midsagittal images. Immediately afterwards, a 3D volume was acquired at the maximum sweep angle (90°) to confirm probe alignment and allow offline reconstruction. During offline review, BPA was measured again in the 3D dataset, selecting the slice providing the most accurate midsagittal view of the pubic symphysis and fetal part. If the difference between 2D and 3D exceeded 5°, the 3D measurement was considered more reliable and used for analysis. This approach minimized errors from slight probe misalignment, incomplete visualization of the symphysis, or tangential cuts of the fetal part.

### 2.3. ECV Procedure

Maternal height and weight were recorded on the day of the procedure. Continuous fetal monitoring was performed for 30–60 min before ECV. Intravenous ritodrine hydrochloride (Pre-Par^®^, Bial, Portugal) (0.2 mg/min) was administered for 30 min prior to the attempt. All procedures were performed in the obstetric operating room by a specialized team comprising an anesthesiologist, a midwife, and at least two of four obstetricians with experience in >1000 ECVs.

Participants were randomized to receive either propofol sedation or spinal anesthesia using a computer-generated sequence that was independent of fetal presentation: (a) Propofol sedation (Propofol Fresenius 10 mg/mL (Fresenius Kabi Deutschland GmbH, Berlin, Germany): continuous infusion targeting plasma concentration 4–6 μg/mL, or (b) Spinal analgesia: Hyperbaric bupivacaine 0.5% (Bupivacaína B. Braun 5 mg/mL; B. Braun Medical, S.A., Barcelona, Spain) combined with fentanyl 20 µg (Fentanest 0.05 mg/mL; Kern Pharma, S.L., Madrid, Spain).

Patients were positioned in 15° Trendelenburg. Depending on fetal lie, forward roll or backflip techniques were used, with a maximum of two attempts. After each procedure, patients were assessed for vaginal bleeding, and fetal well-being was reassessed by ultrasound and cardiotocography (CTG) for 2–4 h. All women were discharged if stable and underwent repeat CTG the following day.

### 2.4. Statistical Analysis

Continuous variables were summarized as median (interquartile range) and categorical variables as count (percentage). Normality and homoscedasticity were assessed using the Shapiro–Wilk and Levene tests, respectively. Student’s *t*-test or Mann–Whitney U test was used for continuous variables, and Pearson’s chi-squared or Fisher’s exact test for categorical variables. A successful ECV was defined as conversion to cephalic presentation immediately after the procedure. Receiver operating characteristic (ROC) curves were used to evaluate the diagnostic accuracy of BPA, with the optimal cut-off determined using the Zweig and Campbell method [18]. Logistic regression was used to evaluate BPA as a predictor of ECV outcome, adjusted for potential confounders (variables with *p* < 0.20 in univariate analysis and those known to be clinically relevant), including type of intervention (spinal vs. propofol). Eight participants with missing BPA values were excluded from analyses involving this variable. All models were therefore fitted on a complete-case basis. All analyses were two-sided with a significance level of 0.05. Statistical analyses were performed using Stata/BE 18.0 (StataCorp, College Station, TX, USA).

## 3. Results

A total of 117 women were included in the analysis: 100 with breech presentation and 17 with transverse lie. The overall success rate of ECV was 61.54% (72/117). In breech presentations ECV success rate was 59% (59/100); meanwhile, it was 76.47% (13/17) in transverse lie.

**Figure 1:**
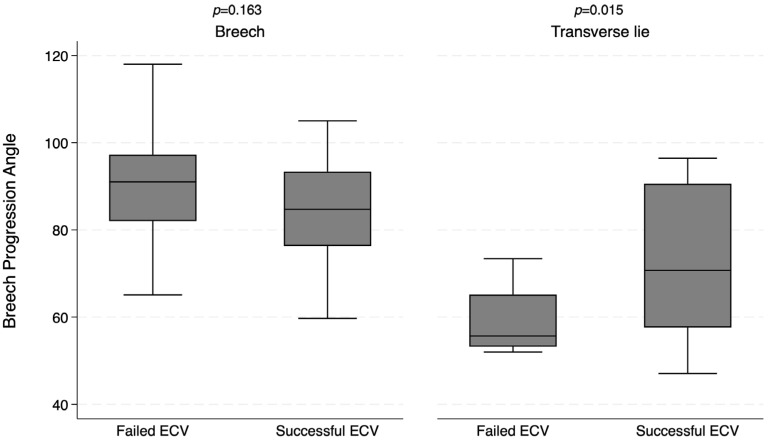
Boxplot of breech progression angle in breech presentation and transverse lie.

Baseline characteristics are shown in Table 1 and Tables S1–S3. Women with transverse lie were older than those with breech presentation (37.3 vs. 32.5 years; *p* = 0.001) and were more likely to receive propofol sedation (94.1% vs. 69.0%; *p* = 0.032). No differences were found in the rest of variables. The median breech progression angle (BPA) was higher in breech than in transverse lie (87.2° vs. 70.2°; *p* < 0.001) (Figure 1).

### 3.1. Association Between BPA and ECV Outcome

Univariate and multivariate analysis is shown in Table 2. In the overall cohort, BPA was not significantly associated with ECV success (OR = 0.97; 95%CI 0.94–1.00; *p* = 0.068). In breech presentations, BPA was independently associated with ECV success (OR = 0.95; 95%CI 0.92–0.99; *p* = 0.018). In transverse lie, no association was observed (OR = 1.08; 95%CI 0.97–1.20; *p* = 0.181).

### 3.2. Predictive Performance of BPA

**Figure 2:**
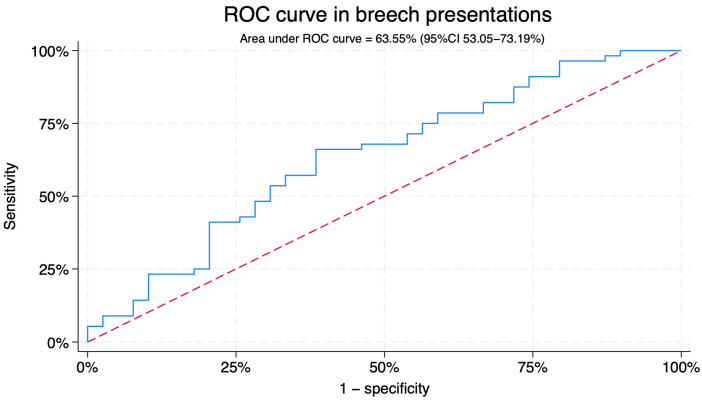
Receiver operating characteristic curve (ROC) for prediction of external cephalic version success in breech presentation. The blue curve represents the observed ROC for breech progression angle. The red diagonal line corresponds to the reference line.

**Figure 3:**
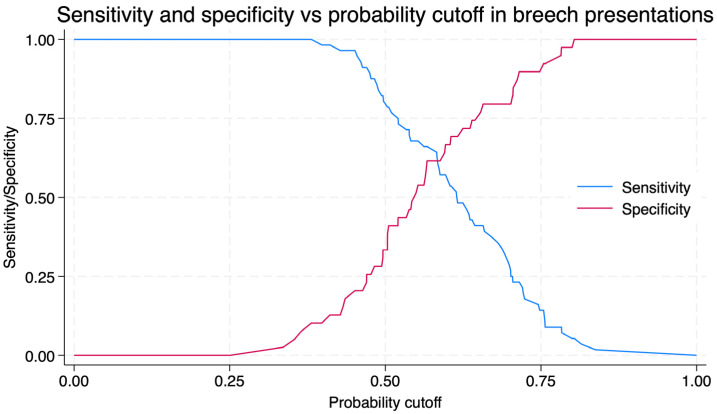
Sensitivity and specificity of the Breech Progression Angle for predicting external cephalic version success in breech presentation, according to different probability cut-offs. The blue curve represents sensitivity, and the red curve represents specificity.

For breech presentations, the area under the ROC curve (AUC) for BPA predicting ECV success was 63.55% (95%CI 53.05–73.19) (Figure 2). Using a cut-off of 89.01°, sensitivity was 61.5% and specificity 66.1%, with a positive predictive value of 75.1% and a negative predictive value of 50.8% (Figure 3). The predicted probability of successful ECV according to the breech progression angle (BPA) in breech presentations is shown in Figure S1.

**Figure 4:**
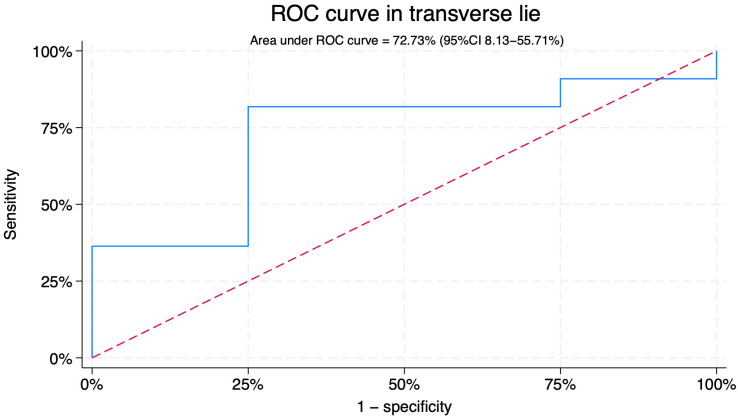
Receiver operating characteristic curve for prediction of external cephalic version success in transverse lie. The blue curve represents the observed ROC for breech progression angle. The red diagonal line corresponds to the reference line.

**Figure 5:**
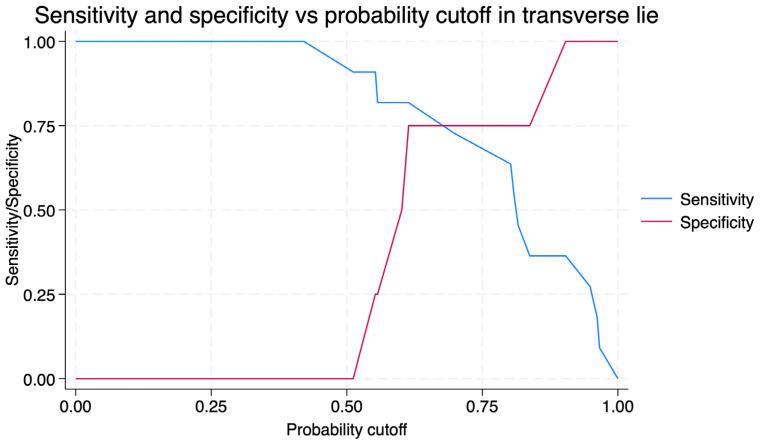
Sensitivity and specificity of the Breech Progression Angle for predicting external cephalic version success in transverse lie, according to different probability cut-offs. The blue curve represents sensitivity, and the red curve represents specificity.

For transverse lie, the AUC was 27.27% (95%CI 8.13–55.71) (Figure 4). With a cut-off of 73.4°, sensitivity was 25.0% and specificity 63.6%, with a positive predictive value of 53.4% and a negative predictive value of 33.7% (Figure 5).

## 4. Discussion

This study explored the potential role of the Breech Progression Angle (BPA) as an ultrasound marker of engagement and its association with the success of external cephalic version (ECV). To our knowledge, this is the first report evaluating BPA in this clinical context. The concept derives from the Angle of Progression used in cephalic labor, which reflects the descent of the fetal head within the birth canal [11,12,14,19].

Youssef et al. proposed the BPA as the breech analogue of the Angle of Progression, demonstrating that it can be reliably measured by transperineal ultrasound and correlates with the degree of breech descent [15,16]. Analogously, a lower BPA may represent a more advanced engagement of the breech within the pelvis, which could theoretically make the ECV procedure more difficult.

In our cohort, lower BPA values were significantly associated with successful ECV in breech presentation, suggesting that less engagement of the fetal breech facilitates the procedure. This finding supports the physiological plausibility of BPA as a potential indicator of fetal station prior to ECV. However, the discriminative performance was modest (AUC 63.55%), indicating that although BPA is statistically associated with outcome, it lacks sufficient accuracy for clinical prediction on its own. The considerable overlap between successful and failed cases further limits its standalone utility in patient counseling or decision-making.

In transverse lie, the presenting part is not engaged within the maternal pelvis, resulting in markedly smaller BPA values. In this subgroup, BPA did not predict ECV success and even showed an opposite, non-significant trend. These findings highlight the anatomical limitations of the measurement in transverse lie and underscore that this analysis should be regarded as exploratory and hypothesis-generating only. Nevertheless, the comparison between breech and transverse presentations provides valuable physiological insight: when the presenting part is not engaged, the BPA is substantially lower, reinforcing its conceptual validity as a sonographic marker of pelvic engagement [20,21].

Prediction of ECV success has long been a clinical challenge [10,22]. Several maternal and fetal factors have been associated with the likelihood of success, including multiparity, non-engagement of the breech, posterior placental location, adequate amniotic fluid, and lower estimated fetal weight [10,22,23]. Nonetheless, no single factor has proven sufficiently accurate to serve as a reliable predictor. Clinical examination to assess engagement is subjective and poorly reproducible [15,16]. Our findings suggest that BPA offers a more objective quantification of engagement, but its predictive value in isolation remains modest.

Our results align with previous literature, as the overall success rate of ECV in our cohort (60.7%) was comparable to that reported in other studies [22,23,24]. Multiparity was associated with higher success rates, consistent with prior findings [22,23,24]. Regarding ultrasound parameters, BPA behaved similarly to other predictors [7]: statistically associated with outcome but insufficiently accurate for clinical decision-making. In breech presentation, the AUC of 63.55% for BPA is comparable to that of other reported sonographic markers of ECV success, such as the amniotic fluid index [7,20].

From a clinical perspective, our findings emphasize the limitations of BPA. Although it provides an objective and reproducible measure of breech engagement—traditionally assessed only by subjective examination—BPA alone does not provide clinically useful information for predicting ECV outcome and should instead be applied within the context of multivariable models that integrate established clinical and sonographic predictors. Future research should investigate whether BPA may have a predictive role in vaginal breech delivery, in analogy to the established value of the Angle of Progression in cephalic labor.

This study has several strengths. To our knowledge, this is the first prospective evaluation of BPA as a predictor of ECV outcome, embedded within a randomized controlled trial with standardized procedures. The use of a rigorous ultrasound protocol, incorporating both 2D and 3D acquisitions, enhanced measurement reliability. The relatively large number of breech presentations allowed adjusted analyses and meaningful comparisons with previous studies. The inclusion of a transverse-lie subgroup, although small, provides valuable exploratory insight into the physiological relationship between pelvic engagement and BPA values.

Nevertheless, interpretation of our results should take into account several limitations. As this was an exploratory and preliminary analysis within a trial primarily designed to compare spinal anesthesia with propofol sedation, the sample size calculation was not tailored to assess BPA, which may have reduced statistical power, particularly for subgroup analyses. The single-center design may limit generalizability, and the very small number of transverse-lie cases weakens the robustness of conclusions for this group. While we did not perform a formal intra-/inter-observer reproducibility analysis in this preliminary report, previous studies have demonstrated excellent repeatability and inter-observer agreement of BPA measurements (ICC 0.83–0.88) [15,16]. We plan to incorporate the same standardized checklist approach prospectively within the ongoing trial to document reproducibility in our cohort. Women in the transverse-lie subgroup were also slightly older, a finding most likely explained by random variation rather than a meaningful clinical association. In addition, the higher proportion of propofol use among women with transverse lie resulted from random imbalance rather than clinical indication, as randomization has not yet fully balanced this variable at this preliminary stage of the trial. Eight participants (6.4%) were excluded because BPA could not be reliably measured, and analyses were conducted on a complete-case basis. Finally, despite adjustment for relevant covariates, residual confounding cannot be entirely excluded.

## 5. Conclusions

This pilot study is the first to evaluate the Breech Progression Angle (BPA) as a predictor of external cephalic version (ECV) outcome. Although lower BPA values were statistically associated with higher rates of successful ECV in breech presentation, the discriminative performance was modest, and BPA alone does not provide clinically useful information for predicting ECV outcome. In transverse lie, BPA lacked predictive value, and the findings should be regarded as hypothesis-generating only. Future studies should focus on developing multivariable models that integrate BPA with other established predictors to refine individualized prediction of ECV success.

## Figures and Tables

**Table 1:** Baseline characteristics. ECV: External cephalic version. BMI: Body Mass Index. CS: cesarean section. AF: Amniotic Fluid.

Variable	Breech *n* = 100	Transverse Lie *n* = 17	*p*
Age (years)	32.5 (28.6–36.6)	37.3 (34–41.1)	0.001
Gestational age at ECV (weeks)	37.6 (37.1–37.7)	37.1 (37–37.7)	0.894
BMI (kg/m^2^)	27 (24.3–30.4)	27.8 (26.8–33.3)	0.184
Nulliparity	62 (62%)	8 (47.1%)	0.245
Previous CS	5 (5%)	2 (11.8%)	0.277
Estimated Fetal Weight (grams)	3002 (2782–3188)	2922 (2839–3092)	0.818
AF Pocket (mm)	50 (42–64.5)	49 (41–65)	0.919
AF Index (mm)	154 (125–182)	155 (137–185)	0.416
Placenta position			0.440
Anterior	41 (41%)	9 (52.9%)	
Posterior	40 (40%)	7 (41.2%)	
Uterine fundus	6 (6%)	1 (5.88%)	
Lateral wall	13 (13%)	0	
Fetal position			<0.001
Transverse lie	0	17 (100%)	
Frank Breech	80 (80%)	0	
Complete breech	16 (16%)	0	
Footling breech	4 (4%)	0	
Analgesia			0.032
Propofol	69 (69%)	16 (94.1%)	
Spinal anesthesia	31 (31%)	1 (5.88%)	
Breech Progression Angle (°)	87.2 (76.7–95)	70.2 (54.4–81.5)	<0.001

**Table 2:** Logistic regression model for breech progression angle predicting ECV outcome. BMI: Body Mass Index.

Variables	Breech			Transverse Lie				
	_crude_OR (95%CI)	*p*	_a_OR (95%CI)	*p*	_crude_OR (95%CI)	*p*	_a_OR (95%CI)	*p*
Breech Progression Angle (°)	0.95 (0.92–0.99)	0.018	0.95 (0.91–0.99)	0.015	1.08 (0.97–1.20)	0.181	1.06 (0.94–1.19)	0.358
Age (years)			0.99 (0.92–1.07)	0.828			1.32 (0.92–1.89)	0.126
BMI (kg/m^2^)			1.03 (0.93–1.13)	0.623			0.80 (0.57–1.14)	0.221
Nulliparity			0.67 (0.27–1.64)	0.379			0.56 (0.01–21.74)	0.759
Propofol			1.07 (0.42–2.72)	0.886			1.00	1.00

## Data Availability

The data presented in this study are available from the corresponding author upon reasonable request.

## Supplementary Materials

The following supporting information can be downloaded at: https://www.mdpi.com/article/10.3390/jcm14207179/s1, Figure S1. Predicted probability of successful ECV according to the breech progression angle (BPA) in breech presentations. BPA: breech progression angle; ECV: External cephalic version. Table S1: Baseline characteristics in breech presentations and transverse lie. ECV: External cephalic version. BMI: Body Mass Index. CS: cesarean section. AF: Amniotic Fluid; Table S2: Baseline characteristics in breech presentations. ECV: External cephalic version. BMI: Body Mass Index. CS: cesarean section. AF: Amniotic Fluid; Table S3: Baseline characteristics in transverse lie. ECV: External cephalic version. BMI: Body Mass Index. CS: cesarean section. AF: Amniotic Fluid.
