# Supplementary material for: Ultrasound Assessment of Breech Engagement: Breech Progression Angle and Prediction of External Cephalic Version Success
Source: J Clin Med. 2025 Oct 11;14(20):7179. doi: 10.3390/jcm14207179 (PMC12564991; doi:10.3390/jcm14207179)
Supplement: Supplementary file 1 [file jcm-14-07179-s001.zip › Table_S1_Supplementary_Total.pdf]

Table S1 – Baseline characteristics in breech presentations and transverse lie. ECV: External cephalic version. BMI: Body Mass Index. CS: cesarean section. AF: Amniotic Fluid.

	Breech N=100	Transverse lie N=17	Total N=117	*p*
**Age (years)**	32.3 (5.71)	37.3 (5.48)	33 (5.92)	**0.001**
**Gestational age at ECV (weeks)**	37.7 (1.09)	37.7 (1.07)	37.7 (1.08)	0.894
**BMI (Kg/m^2)**	28 (4.61)	29.7 (5.18)	28.3 (4.71)	0.184
**Estimated Fetal Weight (grams)**	3016 (331)	2995 (364)	3013 (334)	0.818
**AF Pocket (mm)**	53.3 (16.2)	52.8 (15.2)	53.2 (16)	0.919
**AF Index (mm)**	156 (48.8)	167 (48.3)	158 (48.7)	0.416
**Nulliparity**	62 (62%)	8 (47.1%)	70 (59.8%)	0.245
**Previous CS**	5 (5%)	2 (11.8%)	7 (5.98%)	0.277
**Placenta position**				
Anterior	41 (41%)	9 (52.9%)	50 (42.7%)	
Posterior	40 (40%)	7 (41.2%)	47 (40.2%)	0.440
Uterine fundus	6 (6%)	1 (5.88%)	7 (5.98%)	
Lateral wall	13 (13%)	0 (0%)	13 (11.1%)	
**Fetal position**				
Transverse lie	0	17 (100%)	17 (14.5%)	
Frank Breech	80 (80%)	0	80 (68.4%)	**<0.001**
Complete breech	16 (16%)	0	16 (13.7%)	
Footling breech	4 (4%)	0	4 (3.42%)	
**Analgesia**				
Sedation	69 (69%)	16 (94.1%)	85 (72.6%)	**0.032**
Spinal anesthesia	31 (31%)	1 (5.88%)	32 (27.4%)	
**Breech Progression Angle (º)**	86.4 (11.6)	68.9 (16)	84.0 (13.6)	**<0.001**

Continuous variables are summarized as mean (SD).
Categorical variables are summarized as count (percentage).
