# Supplementary material for: Ultrasound Assessment of Breech Engagement: Breech Progression Angle and Prediction of External Cephalic Version Success
Source: J Clin Med. 2025 Oct 11;14(20):7179. doi: 10.3390/jcm14207179 (PMC12564991; doi:10.3390/jcm14207179)
Supplement: Supplementary file 1 [file jcm-14-07179-s001.zip › Table_S2_Supplementary_Breech.pdf]

Table S2 – Baseline characteristics in breech presentations. ECV: External cephalic version. BMI: Body Mass Index. CS: cesarean section. AF: Amniotic Fluid.

	Failed ECV N=41	Successful ECV N=59	Total N=100	*p*
**Age (years)**	32.3 (5.16)	32.3 (6.1)	32.3 (5.71)	0.963
**Gestational age at ECV (weeks)**	37.8 (1.43)	37.7 (.771)	37.7 (1.09)	0.477
**BMI (Kg/m^2)**	27.6 (4.25)	28.3 (4.85)	28 (4.61)	0.411
**Estimated Fetal Weight (grams)**	2949 (330)	3062 (326)	3016 (331)	0.094
**AF Pocket (mm)**	48.9 (18.3)	56.3 (14)	53.3 (16.2)	**0.024**
**AF Index (mm)**	144 (52.4)	165 (44.6)	156 (48.8)	**0.032**
**Nulliparity**	27 (65.9%)	35 (59.3%)	62 (62%)	0.508
**Previous CS**	0 (0%)	5 (8.47%)	5 (5%)	0.056
**Placenta position**				
Anterior	17 (41.5%)	24 (40.7%)	41 (41%)	
Posterior	14 (34.1%)	26 (44.1%)	40 (40%)	0.640
Uterine fundus	3 (7.32%)	3 (5.08%)	6 (6%)	
Lateral wall	7 (17.1%)	6 (10.2%)	13 (13%)	
**Fetal position**				
Frank Breech	32 (78%)	48 (81.4%)	80 (80%)	0.897
Complete breech	7 (17.1%)	9 (15.3%)	16 (16%)	
Footling breech	2 (4.88%)	2 (3.39%)	4 (4%)	
**Analgesia**				
Sedation	28 (68.3%)	41 (69.5%)	69 (69%)	0.899
Spinal anesthesia	13 (31.7%)	18 (30.5%)	31 (31%)	
**Breech Progression Angle (º)**	89.8 (12)	84.0 (10.8)	86.4 (11.6)	**0.015**

Continuous variables are summarized as mean (SD).
Categorical variables are summarized as count (percentage).
