# Supplementary material for: Ultrasound Assessment of Breech Engagement: Breech Progression Angle and Prediction of External Cephalic Version Success
Source: J Clin Med. 2025 Oct 11;14(20):7179. doi: 10.3390/jcm14207179 (PMC12564991; doi:10.3390/jcm14207179)
Supplement: Supplementary file 1 [file jcm-14-07179-s001.zip › Table_S3_Supplementary_Transverse.pdf]

Table S3 – Baseline characteristics in transverse lie. ECV: External cephalic version. BMI: Body Mass Index. CS: cesarean section. AF: Amniotic Fluid.

	Failed ECV N=4	Successful ECV N=13	Total N=17	*p*
**Age (years)**	34.7 (1.81)	38.1 (6.02)	37.3 (5.48)	0.286
**Gestational age at ECV (weeks)**	37.6 (.955)	37.7 (1.13)	37.7 (1.07)	0.823
**BMI (Kg/m^2)**	32.1 (6.11)	28.9 (4.89)	29.7 (5.18)	0.300
**Estimated Fetal Weight (grams)**	2980 (270)	3000 (398)	2995 (364)	0.927
**AF Pocket (mm)**	57.5 (14.5)	51.4 (15.7)	52.8 (15.2)	0.499
**AF Index (mm)**	161 (35.4)	168 (52.7)	167 (48.3)	0.805
**Nulliparity**	2 (50%)	6 (46.2%)	8 (47.1%)	0.893
**Previous CS**	2 (50%)	0 (0%)	2 (11.8%)	**0.007**
**Placenta position**				
Anterior	2 (50%)	7 (53.8%)	9 (52.9%)	0.166
Posterior	1 (25%)	6 (46.2%)	7 (41.2%)	
Uterine fundus	1 (25%)	0 (0%)	1 (5.88%)	
**Analgesia**				
Sedation	4 (100%)	12 (92.3%)	16 (94.1%)	0.567
Spinal anesthesia	0	1 (7.69%)	1 (5.88%)	
**Breech Progression Angle (º)**	59.2 (9.7)	72.4 (16.7)	68.9 (16)	0.163

Continuous variables are summarized as mean (SD).
Categorical variables are summarized as count (percentage).
