# Supplementary figures and images for: Ultrasound Assessment of Breech Engagement: Breech Progression Angle and Prediction of External Cephalic Version Success
Source: J Clin Med. 2025 Oct 11;14(20):7179. doi: 10.3390/jcm14207179 (PMC12564991; doi:10.3390/jcm14207179)

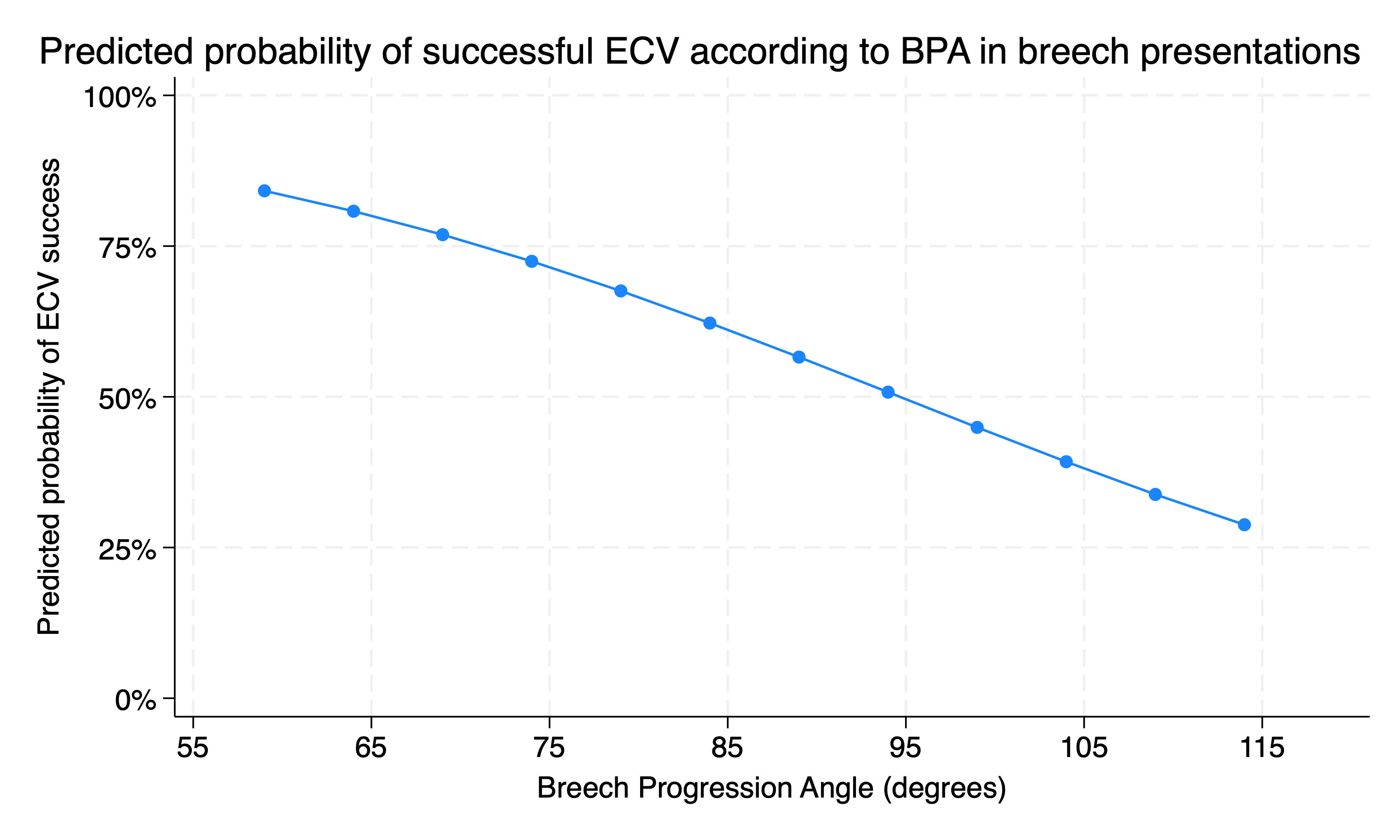

Supplement: Supplementary file 1 [file jcm-14-07179-s001.zip › Figure_S1.tif]
